# Signaling mechanisms and their regulation during in vivo or in vitro maturation of mammalian oocytes

**DOI:** 10.1186/s12958-022-00906-5

**Published:** 2022-02-24

**Authors:** Patrycja Strączyńska, Krzysztof Papis, Emilia Morawiec, Michał Czerwiński, Zdzisław Gajewski, Anita Olejek, Anna Bednarska-Czerwińska

**Affiliations:** 1 Department of Gynecology, Obstetrics and Oncological Gynecology in Bytom, Medical University of Silesia, Katowice, Poland; 2 Faculty of Medical Sciences in Zabrze, Medical University of Silesia, Katowice, Poland; 3 Gyncentrum Fertility Clinic, Katowice, Poland; 4 Center for Translational Medicine, Warsaw University of Life Sciences, Warsaw, Poland; 5 nOvum Fertility Clinic, Warsaw, Poland; 6 Department of Microbiology, Faculty of Medicine in Zabrze, University of Technology in Katowice, Katowice, Poland

**Keywords:** Oocyte maturation, cAMP, cGMP, C-type Natriuretic Peptide, In vitro fertilization

## Abstract

In vitro fertilization (IVF) is currently one of the most effective methods of infertility treatment. An alternative to commonly used ovarian hyperstimulation can become extracorporeal maturation of oocytes (in vitro maturation; IVM). Fertilization and normal development of the embryo depends on the cytoplasmic, nuclear and genomic maturity of the oocyte. The microenvironment of the ovarian follicle and maternal signals, which mediate bidirectional communication between granulosa, cumulus and oocyte cells, influence the growth, maturation and acquisition of oocyte development capability. During oogenesis in mammals, the meiosis is inhibited in the oocyte at the prophase I of the meiotic division due to the high cAMP level. This level is maintained by the activity of C-type natriuretic peptide (CNP, NPPC) produced by granulosa cells. The CNP binds to the NPR2 receptor in cumulus cells and is responsible for the production of cyclic guanosine monophosphate (cGMP). The cGMP penetrating into the oocyte through gap junctions inhibits phosphodiesterase 3A (PDE3A), preventing cAMP hydrolysis responsible for low MPF activity. The LH surge during the reproductive cycle reduces the activity of the CNP/NPR2 complex, which results in a decrease in cGMP levels in cumulus cells and consequently in the oocyte. Reduced cGMP concentration unblocks the hydrolytic activity of PDE3A, which decreases cAMP level inside the oocyte. This leads to the activation of MPF and resumption of meiosis. The latest IVM methods called SPOM, NFSOM or CAPA IVM consist of two steps: prematuration and maturation itself. Taking into account the role of cAMP in inhibiting and then unblocking the maturation of oocytes, they have led to a significant progress in terms of the percentage of mature oocytes in vitro and the proportion of properly developed embryos in both animals and humans.

## Background

In sexually matured mammals, recruitment, activation and growth of dormant ovarian follicles is triggered via c-Kit/kit ligand-phosphoinositide 3-kinase (PI3K)—phosphatase and tensin homologue (PTEN)- protein kinase B (AKT) signaling pathway. In the mouse ovary, activated AKT migrates to the oocyte’s nucleus to suppress transcriptional activity of forkhead box O3 (FOXO3) considered along with a serine/threonine kinase, known as mammalian target of rapamycin (mTOR) as a main follicle/oocyte dormancy factor. A subsequent follicle growth requires specific overcoming an inhibitory MTS 1/2 (macrophage stimulating protein 1/2) (or Hippo) signaling pathway [reviewed thoroughly in: 47,116]. These finally result in the growth and differentiation of the ovarian follicles from the primordial follicle stage, through primary, secondary, up to the Graafian follicle.

In vitro fertilization (IVF) is currently one of the most effective methods of infertility treatment. The use of controlled ovarian hyperstimulation (COH) to induce the growth and development of a higher number of ovarian follicles, thereby obtaining more mature egg cells, has led to an increase in the pregnancy rate per cycle. However, COH may be the cause of one of the most serious complications associated with in vitro fertilization, which is ovarian hyperstimulation syndrome (OHSS), potentially life-threatening depending on its severity. Patients with polycystic ovary syndrome (PCOS) are at higher risk of OHSS and this further increases with the use of chorionic gonadotropin (hCG), which is necessary to initiate oocyte maturation and to induce synchronized ovulation [75]. PCOS affects 6–10% of women worldwide [53].

The use of GnRH instead of hCG, due to its downstream stimulation of ovulation by means of endogenous LH physiological activity significantly limits the risk of OHSS complications [31] and has become the gold standard for triggering oocyte maturation and ovulation in all IVF cycles [48]. However, in vitro maturation of oocytes as a method alternative to controlled ovarian hyperstimulation is still seriously considered [18, 111] In this case, immature oocytes at the germinal vesicle (GV) stage, inhibited in the prophase of the first meiotic division, are collected from the growing antral follicles before maturation begins. Immature oocytes released from the factors inhibiting meiosis (oocyte maturation inhibitor, OMI) in the follicular fluid [7, 65, 106] have the ability to spontaneously resume and complete reductive cell division under laboratory conditions [84]. Most studies on the regulation of oocyte developmental competence are carried out extracorporeally. Therefore, IVM is both a research target and a valuable tool for assessing the factors influencing the developmental competence of the oocyte [1, 7, 120].

The optimal use of the factors affecting the resumption of meiosis and the study of the mechanisms of their mutual regulation remain in the area of interest of both medically assisted procreation and biotechnology of animal reproduction, especially domestic cattle, in which nearly a million embryos are produced annually in vitro using IVM methods [51].

### Ovarian follicle and oocyte development

The process of oogenesis begins during fetal life and continues until the end of the female's reproductive period. During oogenesis, female gametes are produced from primordial germ cells. In human, around 24th day of gestation, primordial germ cells form in the wall of the yolk sac and migrate to the gonadal ridges around the 5th week of gestation. After reaching the primary gonad, cells repeatedly divide mitotically, forming oogonia. They then differentiate into primary oocytes, which undergo the first meiotic division and are arrested at the prophase I. At birth, there are approximately 2 million oogonia in the human female gonad. As a result of activation and growth, only about 400 of them will reach the maturation and release stage [45]. The remaining primordial follicles will undergo apoptosis or atresia at various stages of growth. In most vertebrates, including humans, during oogenesis, when primordial follicles are formed, meiosis is arrested at prophase I. Inhibition of meiotic division at this stage allows the oocyte to pass through the growth phase, during which proteins and mRNA, necessary for the proper course of maturation and the initial stages of embryonic development, are accumulated in the cytoplasm [52]. An oocyte arrested at prophase I contains a large nucleus (also called an embryonic or germinal vesicle) with a visible nucleolus [17]. Folliculogenesis is a separate sub-process that accompanies and supports oogenesis. Recruitment, activation and growth of dormant ovarian follicles is triggered via c-Kit/kit ligand-PI3K-PTEN-AKT pathway, leading to suppressing activity of FOXO3 and mTOR, the main follicle dormancy factors [47, 116], resulting in the growth and differentiation of the ovarian follicles from the primordial follicle stage, through primary, secondary, up to the Graafian follicle. Differentiation of granulosa cells (GCs), cumulus oophorus cells (CCs) and corona radiata cells take place in each cycle in the growing and maturing follicle. The complex consisting of these three structures and the oocyte is called the cumulus oocyte complex (COC) [26, 52, 84].

Maturation begins with the germinal vesicle stage, through the germinal vesicle breakdown (GVBD), metaphase I (MI), and ends with metaphase II (MII). Reaching the MII by the oocyte is necessary for the proper fusion of gametes during oocyte fertilization and for chromosome reorganization during zygote formation. However, each of the earlier stages is equally important as it allows cell to gradually attain an optimal state of developmental competence that demonstrates the maturity of the egg cell and its ability to develop properly after fertilization.

The fertilization and normal development of the embryo depends on the cytoplasmic, nuclear and genomic maturity of the oocyte [50, 58, 120]. Nuclear maturity is achieved during the resumption, course and completion of the first meiotic division and the transition to the second division, which in mature oocyte will be arrested at metaphase II. Cytoplasmic maturity consists of all changes in the oocyte cytoplasm, including the accumulation of polyadenylated maternal RNA and proteins necessary for gamete fusion during fertilization and mitotic divisions of the embryo under the control of oocyte mRNA until the embryonic genome is gradually activated [52, 73, 120].

### The role of cumulus oophorus cells and selected proteins in the oocyte cytoplasmic maturation

During the early growth phase, the oocyte secretes glycoproteins that condense around it to form a translucent, acellular layer called zona pellucida (ZP). It defines the zone that separates the oocyte from granulosa cells [6, 85]. The smallest follicles, called primordial follicles, consist of a single layer of flat granulosa cells. During the ovarian follicle development, the shape and number of cells change, and an eccentrically located cavity (follicular antrum) is formed among the cells of the granulosa layer. The antrum is filled with follicular fluid produced by granulosa cells [89, 90]. Increased fluid volume enlarges the follicular cavity and shifts the oocyte towards its peripheral part [30, 38, 42, 52, 63]. The main requirement for the oocyte to achieve cytoplasmic maturation is its normal contact with the granulosa cells of the follicle, maintained by transzonal projections (TZPs) [30, 59, 66], which penetrate through the zona pellucida reaching the oolemma surface [42]. One form of TZPs ending that enables bidirectional exchange of low molecular weight substrates between the oocyte and granulosa cells is the gap junction (GJ) [59]. The gap junctions also create numerous connections between adjacent granulosa cells, which is the basis for the formation of an extensive network of intercellular communication between the somatic cells of the ovarian follicle. The network of these connections is also very important because the layer of granulosa cells is completely nonvascular [5].

The gap junctions are composed of proteins from the connexin family [5, 77]. The main role in folliculogenesis is played by connexin 43 (Cx43), present on the membrane of both cumulus oophorus/granulosa cells and oocyte as well [35, 40] whose expression increases with the growth of the follicle in response to FSH [77, 98, 99], and connexin 37 (Cx37) responsible mainly for communication between the oocyte and granulosa cells [3, 61, 110]. It has been shown that well-functioning gap junctions are necessary to achieve cytoplasmic and nuclear maturation by the oocyte [15].

Studies of defects in the development of the oocyte and granulosa cells in mice diagnosed with mutations in the genes encoding these proteins showed that the lack of the Cx43 stops the growth of the ovarian follicle during the early stages of development and blocks meiosis [3, 77]. On the other hand, disruption of the gap junctions by switching off the Cx37 gene in mice resulted in the presence of decondensed chromatin in oocytes, typical for the GV stage and the interphase microtubule system, and consequently the inability to resume meiosis [15].

The low quality of oocytes is one of the key factors limiting female fertility. The microenvironment of the ovarian follicle and maternal signals via granulosa and cumulus cells have the most important influence on the growth, development and acquisition of oocyte development competence. The interaction between the oocyte and the granulosa cells is bidirectional. The oocyte secretes growth factors, recently referred to as Oocyte Secreted Factors (OSF) [37] or Oocyte Derived Growth Factors (ODGF) [117, 119], which directly affect adjacent follicular cells, influencing processes within the ovary, including folliculogenesis. This was demonstrated by pioneering studies by Nekola and Nalbandov (1971), in which premature luteinization of granulosa cells in rabbit antral follicles was observed after COC aspiration [93]. Granulosa cells grown in follicle together with the oocyte were less luteinized than those cultured without oocytes [28, 76]. The proposed hypothesis, according to which the oocyte secretes the factors preventing follicle luteinization, was confirmed by studies conducted in the 1990s [11, 109]. On the contrary to the granulosa and cumulus cells, oocytes are not equipped with some important receptors, e.g. for LH [115], so it is thanks to the network of intercellular connections that key signaling molecules, such as e.g. cGMP or cAMP, reach the oocyte [5]. In recent years, it has been confirmed that the oocyte has the ability to regulate the functions of granulosa and cumulus cells, and that there is a two-way axis of communication between the oocyte and somatic cells in mammals [36, 89].

Factors belonging to the TGF-β protein family play an important role in oocyte-somatic cell communication. It consists of about 35 proteins, including the TGF-β group, activin/inhibin, GDF, BMP and anti-Müllerian hormone (AMH) [13, 14, 49, 81]. One of the most important growth factors is GDF-9, classified as a paracrine factor. It has been shown that its task is to lead to the full oocyte developmental competence. The oocytes of GDF-9 knockout mice, despite reaching the appropriate size, do not acquire meiotic competence [14].

Another paracrine factor belonging to the TGF-β family produced by the oocyte is GDF-9B, also called bone morphogenetic protein 15 (BMP-15). It is present in primary follicles in mice, rats, sheep and humans. It has been shown that both BMP-15 and GDF-9 are present in growing follicles, which indicates their important role in the regulation of follicular development [36]. Both proteins are responsible for activating signaling pathways in the cumulus oophorus cells. They participate in the differentiation of cumulus cells by regulating key genes and cellular processes [36]. A deletion in the BMP15 or GDF9 genes directly affects fertility [81]. In a study using a mouse model, it was demonstrated that both ovulation and fertilization were limited in BMP15^(−/−)^ females, while GDF9^(−/−)^ mice were characterized by suppressed follicular growth in the primary follicle stage and were also sterile. Based on the above studies, BMP-15 and GDF-9 proteins are considered fertility regulators [54]. It is also known that abnormal GDF9 expression is associated with polycystic ovary syndrome, while mutations of both GDF9 and BMP15 are associated with ovarian failure [80, 81].

BMP-15 and GDF-9 proteins significantly influence the in vitro maturation of oocytes. Both are detected in the follicular fluid [54, 55]. The addition of BMP-15 and GDF-9 to the medium for extracorporeal maturation increases the developmental competence of oocytes [114].

### Regulation of oocyte meiosis with intercellular communication.

The meiotic and developmental competences of oocytes are acquired in a gradual and sequential manner during folliculogenesis. The interaction of the oocyte with its companion somatic cells is essential for its growth and development. Two cell signaling molecules are involved in the regulation of oocyte meiosis at the level of intercellular communication: cyclic adenosine monophosphate (cAMP) and cyclic guanosine monophosphate (cGMP). They play an important role in maintaining the meiosis inhibition of the oocyte, regulating its maturation by blocking or initiating meiotic processes. Resumption of meiosis is possible due to the active maturation promoting factor (MPF) [16, 65]. MPF is a heterodimer consisting of two subunits: regulatory cyclin B and catalytic CDK1 [2, 79].

As the follicle grows, the oocyte grows to its optimal size and increases CDK1 expression, thus approaching the pre-ovulation stage. When CDK1 is present in sufficient quantity, it is activated by dephosphorylation of tyrosine residues (Tyr 14 and Tyr 15) and binding to cyclin B [70]. Resumption of meiosis is possible in response to hormonal induction or isolation of the egg cell from the ovarian follicle and subsequent in vitro culture [17]. During the meiosis resumption, active MPF leads to the lamin phosphorylation and depolymerization. One of the first visible signs of meiosis resumption is the nuclear envelope breakdown and nucleolus disappearance [65]. In the literature, this phenomenon is referred to as germinal vesicle breakdown (GVBD). At the same time, there are changes in the chromatin (condensation) leading to the appearance of spatially arranged chromosomes in the form of a metaphase plate. At this stage, known as the metaphase of the first meiotic division (MI), there is a transient decline in MPF activity due to the proteolytic degradation of cyclin B [23, 65]. After completion of the first meiotic division, the spindle and the metaphase plate of the second meiotic division (metaphase II; MII) are formed. It is possible due to the continuous synthesis of cyclin B and its connection with CDK1, thanks to which the activity of MPF increases again [2].

As previously mentioned, after COC removal from a preovulatory follicle, meiosis resumes spontaneously in the oocyte. In the follicle, a key role in the oocyte arrest at prophase I of meiotic division is played by the oocyte maturation inhibitor (OMI) [16, 70], the presence of which was postulated in the mid-1970s [106]. OMI and/or mediating signaling molecules such as cGMP or cAMP reach the oocyte from the mural granulosa and cumulus cells through gap junctions and via the follicular fluid.

It is known that the maintenance of oocyte meiotic arrest in prophase I before an increase in the level of luteinizing hormone (LH), depends on the high cAMP concentration in the egg cell. On the other hand, the decrease in the cAMP level allows meiosis to resume [23], initiating the resumption of meiosis. Gs protein activity plays an important role in maintaining a high level of cAMP in the germinal vesicle oocytes. G protein is an integral part of the oocyte plasma membrane composed of αβγ subunits. Active G protein (Gs) is present in a form that has been dissociated into the βγ and Gα-GTP dimer. It has been confirmed that the injection of a Gs inhibitor or the dominant inactive form of G protein into the oocyte causes the resumption of meiosis in frogs [34] and humans [22]. Gs stimulates at least one form of adenylate cyclase (type 3) to produce cAMP. Gs is in turn stimulated by the G-protein coupled receptor 3 (GPR3), which is an adenylate cyclase activator. If any factor in this pathway is eliminated in the oocyte within the follicle, maturation will occur spontaneously [68, 69]. GPR3 permanent expression is required to maintain meiotic arrest in the mouse oocyte [69].

The activity of gap junctions through which cAMP enters the oocyte from the granulosa cells plays an important role in the regulation of meiosis. It was shown that a 5-h incubation of rat oocytes with carbenoxolone (CBX), a selective blocking agent of gap junctions within the ovarian follicle, promoted the maturation of almost all oocytes. Oocyte maturation induced by CBX was accompanied by a significant decrease in cAMP concentration, reportedly not associated with increased phosphodiesterase type 3A (PDE3A) activity [95]. This research confirms earlier observations on key role of gap junctions in cAMP transmission from granulosa and cumulus cells into the oocyte. High level of cAMP is an important signaling pathway responsible for low MPF activity, thus maintaining meiotic arrest of oocyte [19–21].

### The role of cyclic AMP in the oocyte and the luteinizing hormone-induced decrease in the synthesis of cyclic GMP

One of the main regulators of meiotic division is the previously mentioned maturation promoting factor. Cyclic AMP maintains a meiosis inhibited state in prophase I through protein kinase A (PKA). It is responsible for the high level of CDK1 phosphorylation at three amino acid residues: Threonine 161 (Tre 161), Tyrosine 14 (Tyr 14), Tyrosine 15 (Tyr 15), which allows the MPF CDK1 subunit to remain inactive. MPF activity is regulated by Wee1/Myt1 kinases and Cdc25 phosphatases [79]. Activation of CDK1 requires dephosphorylation of tyrosine residues (Tyr 14 and Tyr 15) by means of Cdc25b isoform activity [35, 62] and conjunction with cyclin B. Resumption of meiosis is dependent on the cyclin-1 dependent kinase (CDK1, p34cdc2). Cyclin-1-dependent kinase activators are phosphatases with dual specificity: Cdc25a, Cdc25b, Cdc25c, which are responsible for the regulation of the cell cycle in mitosis and meiosis. Mice lacking Cdc25b provide the first genetic model for studying the mechanisms regulating prophase arrest in vertebrates. Lincoln et al. [62] confirmed that female Cdc25^(−/−)^ mice were sterile and the oocyte of Cdc25^(−/−)^ mice was inhibited in prophase I, which resulted in low MPF activity. Microinjection of mRNA Cdc25b into the Cdc25b^(−/−)^ mice caused an increase in MPF activity and resumption of meiosis [62]. The phosphorylation status of the key tyrosine residues (Tyr 14 and Tyr 15) depends on the balance between the activity of Wee1/Myt1 kinases and Cdc25 phosphatase [44, 60, 74]. Mammalian oocytes in Graafian follicles are maintained in prophase I of the first meiotic division until the preovulatory increase in LH [67]. Resumption of meiosis is possible due to processes triggered by the binding of LH with a specific transmembrane receptor (LHR) present on granulosa and cumulus cells. LH surge causes a transient, many folds rise cAMP synthesis in granulosa cells, resulting in a local EGFβ family growth factors (amphiregulin, epiregulin, betacellulin) activation [39, 46, 87] and decrease of cGMP synthesis and/or increase of its hydrolysis, accordingly reducing the flow of cGMP from the granulosa cells into the oocyte. The decrease in cGMP attenuates the inhibition of hydrolytic PDE3A activity, which leads to a lowering of the cAMP level in the oocytes, resulting in the resumption of meiosis.

On the other hand, LH surge triggers an alternative, well defined mechanism based on a breakdown of communication between the oocyte and somatic cumulus and granulosa cells ceasing influx of cAMP molecules into the oocyte [20, 29, 83, 95]. This gating effect of LH was shown to be comprised of two consecutive steps: an immediate transient upregulation of the phosphorylation state of Cx43 and a later reduction in the CX43 protein level by transcriptional or translational modifications [40, 41, 56]. This breakdown of gap-junctional communication possibly results in resumption of meiosis (reviewed in: [25]). Taken together cAMP signaling as well as cGMP signaling are involved in the control of meiotic maturation. A decrease in the activity of any of the cyclic nucleotides is a signal for the oocyte to resume meiotic maturation [78, 107].

Cyclic nucleotide phosphodiesterases (PDEs) are a group of enzymes that hydrolyze cyclic nucleotides in a variety of cell types, including granulosa cells and oocytes. PDE isoenzymes are capable of hydrolyzing cAMP, cGMP, or both. PDE3A is one of the most important cAMP-hydrolyzing enzymes, which in turn is inhibited by cGMP [43]. It has been proved that cGMP inhibits the hydrolytic activity of cAMP phosphodiesterase in oocyte lysate [9, 10]. Moreover, it has been reported that cGMP diffuses through the gap junctions into the oocyte, where it inhibits cAMP hydrolysis being therefore responsible for maintaining of meiosis arrest [78, 107]. More recent studies have shown that in growing and preovulatory follicles, granulosa cells secrete into the follicular fluid, a C-type natriuretic peptide (NPPC, also known as CNP) capable of binding to cumulus cells membrane natriuretic peptide receptor 2 (NPR2, also known as guanylate cyclase B), belonging to the guanylate cyclase receptor family. The CNP/NPR2 complex, acting actively as guanylate cyclase, increases the concentration of cGMP both in cumulus cells and, indirectly, in the oocyte, maintaining meiosis arrest [27, 119]. CNP has been identified as a meiosis inhibitory peptide in mouse [119], rat [118], pig [93] and cattle [32] oocytes. The mechanism of CNP action and its structure corresponds to the description of the oocyte maturation inhibitor (OMI) detected in the follicular fluid by A. Tsafriri in the 1970s [106].

Shuhaibar et al., [97] using mouse follicles to monitor cGMP levels in real time from LH exposure, noted that within 1 min of LH exposure, cGMP levels in granulosa cells began to decline. After 20 min, the cGMP concentration had decreased by more than 20-fold and was low in the entire follicle. As mentioned above, the concentration of cAMP in the oocyte remains high due to the inhibitory effect of cGMP on PDE3A [97]. LH-induced decrease in cGMP synthesis in granulosa and follicular cells, and consequently also in the oocyte, unblocks phosphodiesterase activity, which hydrolyzes cAMP, allowing meiosis to resume. This effect, at the oocyte level, can be blocked by an exogenous, specific PDE3A inhibitor (e.g. milrinone) [78]. In summary, the constant synthesis of CNP in granulosa cells and the catalytic activity of the CNP/NPR2 complex are the main source of cGMP both in the somatic cells of the follicle and, indirectly, in the oocyte [39, 119]. The signal in the form of LH surge, acting through a specific membrane LH receptor located in outer granulosa and cumulus cells, initiates a cascade of reactions leading to the meiosis resumption [5, 108]. LH, acting via PKA, activates phosphodiesterase 5 that degrades cGMP, while limiting the activity of NPR2. Thus, inhibiting cGMP synthesis causes a rapid decrease in the cGMP level in the outer layers of granulosa cells. The LH surge during each reproductive cycle induces a reduction in CNP activity, which results in a decrease in the cGMP level in the granulosa cells and indirectly in the oocyte. Due to this, PDE3A is activated, causing a decrease in the level of cAMP inside the oocyte, leading to the activation of MPF and, consequently, to the resumption of meiosis [39].

**Fig. 1:**
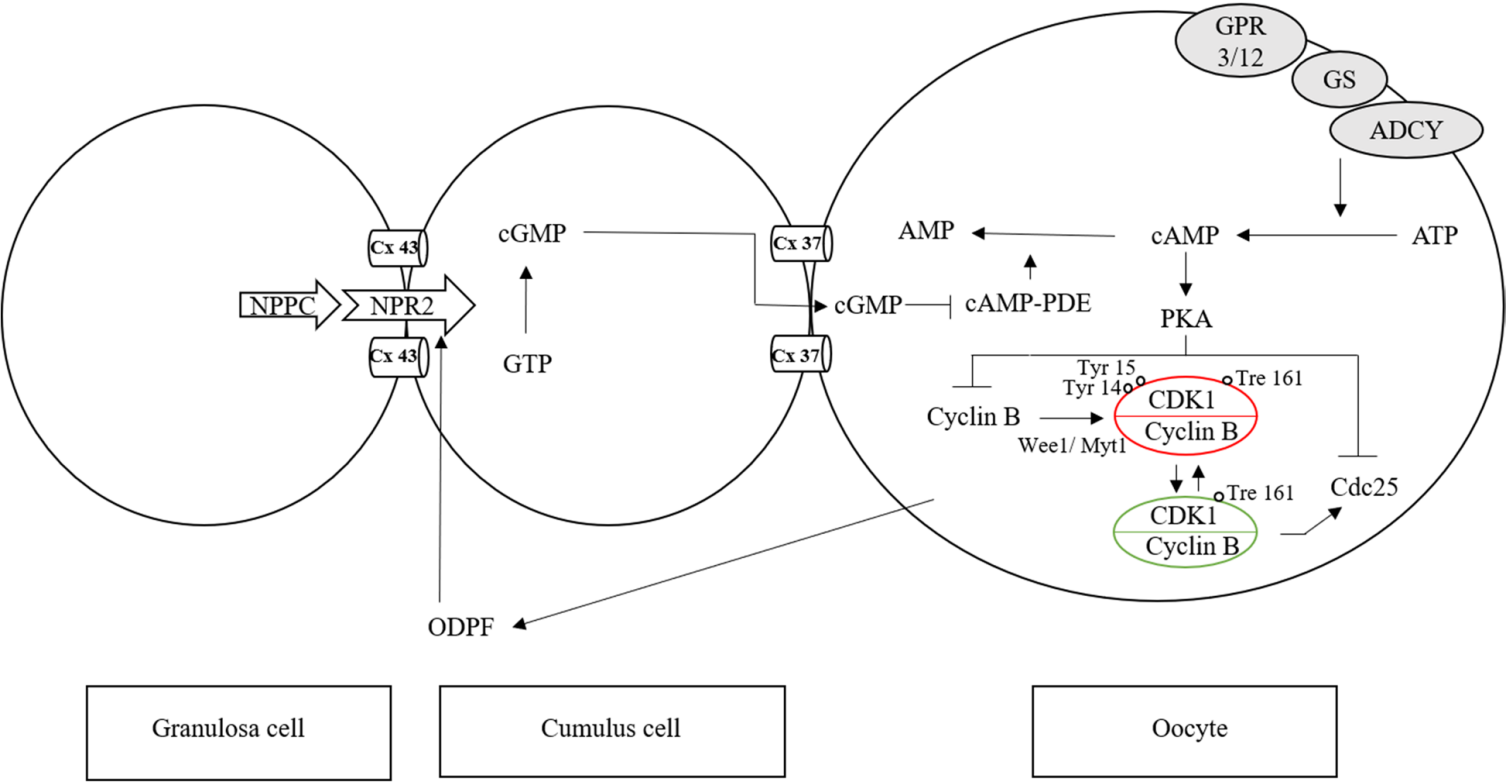
Scheme of maintenance of meiotic arrest in mammals. cAMP inside the oocyte is produced by activation of the G-protein coupled receptor (GPR) 3 and GPR 12 activating adenylate cyclase. Natriuretic peptide precursor C (NPPC), produced by the granulosa cells, stimulates the production of cGMP via specific NPR2 receptor. cGMP diffuses through the gap junctions, inhibiting phosphodiesterase (PDE) activity and cAMP hydrolysis, maintaining a high level of cAMP in the oocytes. cAMP-dependent protein kinase regulates the activity of the maturation promoting factor MPF (CDK1-cyclin B). The high level of cAMP results in CDK1 phosphorylation and inactivation of the CDK1-cyclin B complex. Oocyte-derived paracrine factors (ODPFs) maintain meiosis arrest by stimulating NPPC and NPR2 expression and cGMP accumulation based on [82, 119] modified

The above studies allowed for the development of a logical model showing a balanced state of meiosis inhibition as the end result of the cascade reactions of messenger molecules initiated at the ovarian follicle level by the catalytic activity of the CNP/NPR2 complex (Fig. 1). The disturbance of this balance, corresponding to the resumption of meiosis, finds its physiological source in the form of the LH surge.

Recently, Xi et al. identified an additional CNP-induced meiosis inhibition/resumption mechanism exclusively expressed in bovine oocytes. They showed that bovine NPR2, a CNP receptor, is located not only in cumulus cells but also in the oocyte membrane. They proved that CNP, due to the active NPR2, stimulates the synthesis of cGMP inside the oocyte, cooperating with a parallel pathway mediated by cumulus cells, being however, independent of it. It can be assumed that the catalytic activity of the CNP/NPR2 complex, by providing the cell with endogenous cGMP, slows down the activation of the hydrolytic activity of PDE3A. Therefore, it delays the decrease in the cAMP level, important for the resumption of meiosis, especially since the LH receptor responsible for the weakening of guanylate cyclase (CNP/NPR2) activity in granulosa cells is absent in the oocyte.

The adequate cGMP level maintains meiosis inhibition in bovine oocytes, while expression of NPR2 in cumulus cells and in the oocyte membrane is synergistically regulated by estradiol and oocyte-secreted factors (OSFs). A high level of cAMP in the oocytes is essential to maintain preovulatory oocytes in a state of meiotic inhibition, and a non-physiological, too rapid decrease in the cAMP level may result in asynchronous cytoplasmic and nuclear maturation [37, 108].

### In vitro maturation (IVM) of oocytes

In vitro maturation (IVM) of oocytes allows to obtain egg cells capable of fertilization and normal embryonic development to the blastocyst stage. IVM is used in both humans and animals. The obtained egg cells are used primarily in the procedures of assisted human reproduction, assisted reproduction of farm animals, as well as in the production of transgenic animals and cloning [26, 38, 100]. One of the most important applications of IVM is the assisted reproduction of farm animals, mainly cattle [38, 39]. Extracorporeal maturation is also carried out to a lesser extent in other species, such as pigs, sheep, goats, cats, horses, and camels. The production of bovine embryos using IVM/IVF procedures is estimated at nearly one million embryos per year [86]. However, in spite of optimistic beginnings [26, 57, 104, 105] and initial frequent use, the traditional variants of the IVM procedure routinely employed in both animals and humans turned out not sufficiently effective in terms of the ratio of mature cells, properly developed embryos and the percentage of pregnancies achieved compared to the procedure of hormonal stimulation of patients, ovulation induction, in vivo maturation and oocyte fertilization [38, 88 92]. The principal reason for the poor uptake of human IVM appears to be the lower pregnancy rates compared with conventional IVF [12]. This lower efficiency manifests at multiple levels: particularly lower metaphase II rates (typically 50–60%), but also lower subsequent embryo development rates [112], and eventually, higher miscarriage rates.

It is well known that IVM may be an effective treatment for infertility in patients with polycystic ovary syndrome (PCOS). These patients spontaneously produce an excessive number of antral follicles that can be punctured during OPU but the collected oocytes must undergo the procedure of extracorporeal maturation. In addition, PCOS patients are more susceptible to developing ovarian hyperstimulation syndrome (OHSS), a complication that occurs in a small proportion of hormonally stimulated patients, potentially life-threatening. Another important application of IVM is in oncofertility procedures, allowing to preserve the fertility of women suffering from cancer and requiring immediate aggressive chemo- or radiotherapy. Such patients are at risk of iatrogenic secondary gonadotoxicity, which often results in infertility. The procedure of extracorporeal maturation of oocytes allows for immediate collection of immature oocytes without the use of time-consuming hormonal stimulation, and therefore without an increase in the estrogen level, unacceptable for certain types of cancer [18, 39].

After in vitro maturation, the oocytes can be frozen and, after the cancer is cured, used in an IVF procedure giving a real chance of pregnancy. However, the incomplete effectiveness of traditional IVM methods hinders these applications. The standard, most frequently used IVM procedure is to remove immature cumulus-oocyte complexes (COCs) from antral follicles and culture them until the MII stage is reached. As described above, most oocytes undergo a spontaneous resumption of meiotic maturation after the mechanical extraction of the COC from the ovarian follicle [84]. One of the first methods of delaying spontaneous resumption of meiosis in vitro was the maturation of oocytes in follicular fluid [8] or in media containing the supplement of follicular fluid [72, 121]. There was a slight slowdown in meiotic maturation and a decrease [8] or a marginal increase in the ratio of developing bovine embryos [71] reported.

Subsequent attempts to improve the IVM system included, among others, the introduction of pharmacological modification of cAMP levels. The use of a cAMP analogue (dbcAMP, dibutyrylcyclicAMP) was claimed successful during maturation of porcine oocytes [33]. Similar activity was shown by invasive adenylate cyclase (iAC) [64] and forskolin [96]. The use of these modulators leads to an increase in the cAMP level inside the oocyte to values close to the levels found in vivo during LH surge [52].

The first two-phase IVM systems used phosphodiesterase (PDE) inhibitors, preventing (in the first phase) spontaneous resumption of meiosis after COC removal from the follicle, maintaining moderate cAMP levels by limiting its hydrolysis. For this purpose, isobutylmethylxanthine (IBMX) and specific PDE3A inhibitors—milrinone or cilostamide were used. During the second phase of maturation, the inhibitor was either removed or used at a lower concentration, which allowed for a gradual reduction in the cAMP level enabling the resumption of oocyte maturation stimulated additionally with high doses of FSH [103].

The pre-IVM phase is responsible for the inhibition of a rapid decrease in the level of cAMP, however, resembling the natural conditions in vivo, it may also cause a significant increase in its level [94]. As described in detail above, the level of intracellular cAMP depends on the activity of adenylate cyclase, responsible for the synthesis of cAMP, and the activity of phosphodiesterases that break down cyclic AMP into adenosine monophosphates [37, 108]. IBMX allows to maintain a high concentration of cAMP inside the oocyte and maintain functional communication through the gap junctions in the COC, indirectly delaying the resumption of oocyte maturation in vitro [91, 102]. On the other hand, enrichment of the medium with forskolin stimulates the catalytic subunit of adenylate cyclase [4] causing an increase in the cAMP concentration inside the oocyte [94]. It was assumed that delaying the spontaneous maturation of oocytes while maintaining communication with the cumulus oophorus cells via gap junctions would allow oocytes to accumulate a larger pool of metabolites and to synthesize and store a sufficient amount of nutrients, which may correlate with obtaining embryos with a higher implantation potential in vitro [4, 24, 91, 102].

Experiments performed on animal models have shown that the use of cAMP modulators causes a significant increase in the level of cAMP in oocytes and the maintenance of 60–70% of the oocytes of cattle, sheep and mice in the germinal vesicle phase even after 7 h of incubation compared to 30% of oocytes from the control group [4, 91].

Prolonged maturation is the second step in physiologically simulated in vitro maturation of oocytes. During this stage, COC is subjected to a 24–30-h in vitro culture in a medium enriched with moderate doses of milrinone or cilostamide and follicle stimulating hormone [4]. Specific type 3 PDE inhibitors (milrinone or cilostamide) used in lower doses delay cAMP hydrolysis, allowing oocytes to start the maturation process slowly and gently, which was supposed to have a positive effect on the later development of embryos. About 80% of cattle oocytes matured with the SPOM method reached the metaphase II of meiotic division, and after fertilization—a high ratio of embryos developed to the blastocyst stage [4].

However, a wider use of the two-stage SPOM procedure brought mixed results. Some studies confirmed the beneficial effect of this procedure on the percentage and quality of obtained cattle blastocysts [32, 122]. Other studies revealed no clearly positive effects on the ratio of developing blastocysts [91, 101, 102]. Our own research has shown the possibility of developing better quality embryos (with a higher total number of cells and a higher ICM/TE ratio), but with a lower ratio of developed blastocysts [24, 102]. Recent Danish study has shown that the SPOM method has an adverse effect on the morphology, cell number and development competence of the obtained cattle embryos. Moreover, changes in the expression of many genes essential for the normal embryonic development have been demonstrated [86]. Recently, researchers were interested in the possibility of using CNP as an upstream modulator of cAMP levels in oocytes subjected to modified variants of the two-step methods of maturation of cattle [113], pig (CNP in combination with BNP—B-type natriuretic peptide, specifically present in this species) [122] and also human oocytes [92]. CNP has no direct effect on the cAMP level in cumulus cells or the oocyte, but it induces the production of cyclic guanosine monophosphate in cumulus cells, which is passed through the gap junctions to the oocyte, where it inhibits the activity of phosphodiesterases type 3, which maintains a high level of cAMP in the oocyte [113, 119,119].

The use of CNP as a medium supplement at the pre-IVM stage, referred to in these studies as PMC, allowed for an increase in the percentage of developing good quality bovine blastocysts [113] and parthenogenetic porcine blastocysts [122], while the extended phase of maturation (IVM) allowed for building a high level of ATP in blastomeres, favorable for the further development of embryos [113]. The in vitro oocyte maturation system NFSOM significantly improves the developmental competence of mature oocytes, which translates into increased efficiency of in vitro production of bovine embryos [113].

Very recently, in human settings preclinical effects of CAPA IVM treatment, employing CNP and estradiol supplementation was published by collaborative Belgium – Vietnam research group [111]. After 24 h period of CAPA PMC, a 30 h maturation culture was applied in IVM medium supplemented with FSH and recombinant human amphiregulin (rhAREG). Data shown higher maturation rate of oocytes subjected to CAPA IVM (62 vs 47.9%), higher rate of good quality Day 3 embryos developed per COC (9.1 vs 3.9%) and higher pregnancy rate (58 vs 35%) in comparison to standard IVF, respectively. Particular improvement was achieved for oocytes collected from smaller follicles (< 6 mm) [92]. However, pregnancy rate obtained in clinical trial published by the same group of authors tended to be lower after CAPA IVM in comparison to standard stimulation and IVF (35.2 vs 43.2%) [111]. It may suggest a need of more targeted selection of patients subjected to this kind of specific treatment.

Taken together it seems that the optimization of the method of using CNP as an upstream modulator of the cAMP level in the oocyte will help in the near future to develop such procedures for in vitro maturation of oocytes, which will allow to obtain oocytes with high developmental competence, and after in vitro fertilization—the development of embryos of biological quality as close as possible to that achieved in natural in vivo conditions.

### Summary

In mammalian oocytes, meiosis is inhibited at the prophase I of meiotic division due to the high level of adenosine monophosphate (cAMP), which is responsible for the low MPF activity. The cAMP level is maintained due to the upstream activity type C natriuretic peptide (CNP, NPPC), produced by the granulosa cells, which is responsible for the production of cyclic guanosine monophosphate (cGMP) after binding to a specific NPR2 receptor. cGMP enters the oocyte in order to inhibit phosphodiesterase type 3A (PDE3A), thus preventing cAMP hydrolysis. During each reproductive cycle, the LH surge reduces the activity of the CNP/NPR2 complex, resulting in a decrease in cGMP levels, which in turn leads to the activation of MPF and the resumption of meiosis. The paper presents the latest methods of in vitro maturation of oocytes, imitating the natural, gradual course of this process, referred to as SPOM, NFSOM or CAPA IVM. These methods, taking into account the role of cAMP in inhibiting and then unblocking oocyte maturation, allowed to achieve progress in the development of effective, possibly natural, extracorporeal maturation procedures in animals and in humans.

## Data Availability

All data generated or analyzed during this study are included in this published article.

## Abbreviations

AKT: Protein Kinase B
AMH: Anty-mullerian hormone
ATP: Adenosine-5′-triphosphate
BMP: Bone morphogenetic protein
BMP-15: Bone morphogenetic protein 15
BNP: B-type natriuretic peptide
c-KIT/KITL: C-kit/kit ligand
cAMP: Cyclic adenosine 3′,5′- monophosphate
CBX: Carbenoxolone
CCs: Cumulus cells
Cdc25: Phosphatase Cdc25
CDK1: Cyclin Dependent Kinase 1
cGMP: Cyclic guanosine 3′,5′- monophosphate
CNP: C-type natriuretic peptide
COH: Controlled ovarian hyperstimulation
Cx37: Connexin 37
Cx43: Connexin 43
dbcAMP: DibutyrylcyclicAMP
DNA: Deoxyribonucleic acid
FOXO3: Forkhead box O3
GCs: Granulosa cells
GDF-9: Growth differentiation factor 9
GJ: Gap junctions
GPR3: G-protein coupled receptor 3
Gs: Active protein G
GV: Germinal vesicle
GVBD: Germinal vesicle breakdown
hCG: Human chorionic gonadotropin
iAC: Invasive adenylate cyclase
IBMX: 3-Isobutyl-1-methylxanthine
IVF: In vitro fertilization
IVM: In vitro maturation
LH: Luteinizing hormone
LHR: Luteinizing hormone receptor
MI: Metaphase I
MII: Metaphase II
MTS 1/2: Macrophage stimulating protein 1/2
MPF: Maturation promoting factor
mRNA: Messenger RNA
mTOR: Mammalian target of rapamycin
Myt 1: Myelin transcription factor 1
NFSOM: Natural factor synchronized (in vitro) oocyte maturation
NPPC: Natriuretic peptide precursor type C
NPR2: Membrane natriuretic peptide receptor 2
ODGF: Oocyte derived growth factors
OHSS: Ovarian Hyperstimulation Syndrome
OMI: Oocyte meiosis inhibitor
OSF: Oocyte secreted factors
PCOS: Polycystic Ovary Syndrome
PI3K: Phosphatidylinositol-3-Kinase
PDE3A: Phosphodiesterase 3A
PMC: Pre-maturation culture
PTEN: Phosphatase and tensin homolog
RNA: Ribonucleic acid
SPOM: Simulated physiological oocyte maturation
TGF-β: Transforming growth factor β
Tre 161: Threonine 161
Tyr 14: Tyrosine 14
Tyr 15: Tyrosine 15
TZPs: Transzonal projections
Wee1: Mitosis inhibitor protein kinase
ZP: Zona pellucida
